# Patterns and predictors of co-morbidities in Tuberculosis: A cross-sectional study in the Philippines

**DOI:** 10.1038/s41598-020-60942-2

**Published:** 2020-03-05

**Authors:** Laura V. White, Tansy Edwards, Nathaniel Lee, Mary C. Castro, Naomi R. Saludar, Rugaiya W. Calapis, Benjamin N. Faguer, Nelson Dela Fuente, Ferdinand Mayoga, Nobuo Saito, Koya Ariyoshi, Anna Marie Celina G. Garfin, Juan A. Solon, Sharon E. Cox

**Affiliations:** 1 School of Tropical Medicine and Global Health, Nagasaki University, Nagasaki, Japan; 2 Tropical Epidemiology Group, London School of Hygiene and Tropical Medicine, London, UK; 3 Royal Free Hospital, London, UK; 4 Nutrition Center Philippines, Manila, The Philippines; 5 San Lazaro Hospital, Manila, The Philippines; 6 San Lazaro Hospital PMDT Treatment Center, Manila, The Philippines; 7 Valladolid Health Center, Valladolid, Negros Occidental The Philippines; 8 Bago City Health Center, Bago City, Negros Occidental The Philippines; 9 Institute of Tropical Medicine, Nagasaki University, Nagasaki, Japan; 10 National TB Control Programme, Department of Health, Manila, The Philippines; 11 Faculty of Population Health, London School of Hygiene and Tropical Medicine, London, UK

**Keywords:** Tuberculosis, Malnutrition, Type 2 diabetes, Epidemiology, Epidemiology

## Abstract

Diabetes and undernutrition are common risk factors for TB, associated with poor treatment outcomes and exacerbated by TB. We aimed to assess non-communicable multimorbidity (co-occurrence of two or more medical conditions) in Filipino TB outpatients, focusing on malnutrition and diabetes. In a cross-sectional study, 637 adults (70% male) from clinics in urban Metro Manila (N = 338) and rural Negros Occidental (N = 299) were enrolled. Diabetes was defined as HbA1c of ≥6.5% and/or current diabetes medication. Study-specific HIV screening was conducted. The prevalence of diabetes was 9.2% (54/589, 95%CI: 7.0–11.8%) with 52% newly diagnosed. Moderate/severe undernutrition (body mass index (BMI) <17 kg/^2^) was 20.5% (130/634, 95%CI: 17.4–23.9%). Forty percent of participants had at least one co-morbidity (diabetes, moderate/severe undernutrition or moderate/severe anaemia (haemoglobin <11 g/dL)). HIV infection (24.4%, 74/303) was not associated with other co-morbidities (but high refusal in rural clinics). Central obesity assessed by waist-to-hip ratio was more strongly associated with diabetes (Adjusted Odds Ratio (AOR) = 6.16, 95%CI: 3.15–12.0) than BMI. Undernutrition was less common in men (AOR = 0.44, 95%CI: 0.28–0.70), and associated with previous history of TB (AOR = 1.97, 95%CI: 1.28–3.04) and recent reduced food intake. The prevalence of multimorbidity was high demonstrating a significant unmet need. HIV was not a risk factor for increased non-communicable multimorbidity.

## Introduction

Tuberculosis (TB) remains a global health burden despite advances in diagnosis and treatment and is the leading cause of death from a single infectious agent^1^. Undernutrition is both a risk factor and a consequence of active TB disease and is associated with mortality and adverse outcomes^2,3^. Diabetes is another known risk factor for active TB^4^ and diabetes increases the risk of death and relapse during TB disease^5^ whilst TB can also negatively affect glycaemic control^6^ and may cause the development of diabetes, although this may sometimes be a transient effect^6^. Thus two conditions, both largely nutritional in origin, present a significant challenge to the End TB Strategy targets due to the increasing double burden of under and over nutrition occurring in many high burden, low and middle-income countries^7,8^. For implementation of the END TB strategy, it is recommended that “All persons with TB need to be assessed for nutritional status and receive nutritional counselling and care according to need” and additionally, “all persons with TB should be screened for diabetes” and, “in addition to HIV/AIDS, other co-morbidities and health risks associated with TB require integrated management”^9^. Both diabetes and TB can increase the short and long-term risk of cardiovascular^10^ and respiratory conditions^11^ requiring lifelong management to reduce population health and economic impacts and are recognised as priorities for cost-effective disease control^12,13^. As well as increasing the risk of developing active TB disease, untreated co-morbid undernutrition and diabetes may also increase the duration of infectivity and risk of relapse, thus contributing to ongoing transmission^14^. Undernutrition and anaemia during and after treatment are also likely to affect quality of life and productivity of TB affected households, contributing to a cycle of poverty and risk of catastrophic costs to households; the reduction of which is another goal of the End TB Strategy^8^. However, there are relatively few studies or data specifically documenting the prevalence of these and other co-morbid conditions in persons with TB or of the extent of overlap between them; information needed for the planning of services.

The Philippines, located in the Western-Pacific region is one of the WHO high burden countries for TB and multi-drug resistant TB (MDR-TB) and has the third highest TB incidence of 554/100,000 occurring in the context of a low HIV prevalence, estimated at 2% of incident TB cases, although HIV testing is low in many areas^1^. The prevalence of chronic energy deficiency (BMI < 18.5 kg/m^2^) in Filipino adults was 10% in the most recent National Nutrition Survey completed in 2014^15^. Nationally, the population attributable risk fraction of undernutrition (BMI < 18.5 kg/m^2^) for TB has been estimated at 30%^16^. The prevalence of diabetes in the Philippines is increasing, with the best estimate from 2008 of 7.2%^17^.

There are currently very limited data of the prevalence of co-morbidities in Filipino persons with TB disease. Limited data of diabetes in TB has been identified as a barrier to planning a coordinated response^18^. The Philippine’s Plan of Action to Control Tuberculosis identifies the need to develop guidelines for persons living with diabetes as a vulnerable group, including screening for diabetes in TB, but undernutrition is not mentioned and no TB programme guidelines for its diagnosis and treatment exist^19^. In the general population obesity and in particular central obesity, are common risk factors for diabetes and are important components of various risk scores to detect undiagnosed diabetes. However, the relationship of these in persons with active TB disease is likely to differ, due to TB-associated weight loss.

The aim of this cross-sectional study was to provide a quantitative assessment of non-communicable multimorbidity (co-occurrence of two or more medical conditions)^20^ in TB DOTS outpatients in a rural and an urban setting in the Philippines, focusing on malnutrition and diabetes and on the degree of overlap between co-morbid conditions including HIV infection. The primary objective was to determine the prevalence of moderate or severe undernutrition (body mass index, BMI, <17.0 kg/m^2^) and diabetes (HbA1c ≥ 6.5% or receiving treatment for diabetes) among adults, aged 18 years and above enrolled in our participating TB-DOTS sites.

## Methods

### Study design

A cross-sectional survey of persons enrolled in TB-DOTS clinics (**ISRCTN12506117**).

### Setting

Three TB-DOTS clinics in Metro Manila provided data from an urban setting, including the TB-DOTS and programmatic management of drug resistant TB (PMDT) centres at San Lazaro Hospital, an infectious disease referral hospital and HIV treatment centre, serving a predominantly poor population. Metro Manila is identified as a high HIV category area compared to other areas of the country. Two sites on the island of Negros Occidental provided data from a rural setting.

### Participants

All non-pregnant adults (≥18 years) whose TB-DOTS registration date indicated that TB treatment should be ongoing at each study site, were eligible to participate. This included any patients who had stopped attending the TB DOTS clinic or visited infrequently. Individuals were approached to participate and enrolled at the clinics, or were traced and approached by research nurses in their Barangay (neighbourhood). This was done to attempt to locate all those whose treatment was supposed to be ongoing, to minimize selection bias.

### Outcomes

The primary outcome of interest was co-morbid acute undernutrition (BMI <18.5 kg/m^2^)^21^ and the secondary outcome was diabetes, defined as HBA1c >6.5% or receiving treatment for diabetes at the time of enrolment^22^. However, analyses of undernutrition focussed on moderate and severe undernutrition (BMI <17 kg/m^2^), due to this being a level at which intervention is generally recommended. Other secondary outcomes of interest were moderate or severe anaemia (haemoglobin <11 g/dL)^23^, HIV and reported hypertension.

### Data collection

Trained research nurses interviewed participants and completed all study assessments using structured questionnaires and extracted information recorded on individuals’ National TB Program treatment cards using direct electronic data capture with tablets using Open Data Kit software^24^. Data were uploaded to a secure server daily. Household food security was assessed using the Adapted U.S. Household Food Security Survey Module (US HFSSM)^25^. Research nurses conducted anthropometry, including weight (to the nearest 0.1 kg; Seca 803 Clara Digital Personal Non-Medical Scale) on a flat surface with the patient standing upright and unassisted without shoes. Heights were taken (to nearest 0.5 cm; Seca 216 Mechanical Stadiometer) without shoes or socks, with the patient standing unsupported and positioned fully upright with the lower border of the left orbit and the upper margin of the external auditory meatus horizontal. Waist and hip circumferences were measured (to the nearest 0.5 cm; Seca 201 measuring tape), midway between the uppermost border of the iliac crest and lower border of costal margin with tape parallel to the floor^26^. Fingerprick blood samples were used to obtain haemoglobin (HemoCue 301, Ängelholm, Sweden), HbA1C, C-reactive protein (CRP) (Alere Afinion AS100 Analyzer) and conduct HIV screening (Standard Diagnostics Bioline HIV-1/2 Ag/Ab Combo Rapid Test kits) for those with unknown status and who provided additional consent.

### Sample size

Sample sizes were calculated based on formulae to estimate prevalence with precision^27^. A sample size of 600 (300 urban, 300 rural participants) was determined to provide 90% power to estimate prevalence of undernutrition (BMI < 18.5 kg/m^2^) within ±6% if the true prevalence was between 40–60%, and to estimate the prevalence of diabetes within ±5% if the true prevalence was between 5–15%, or within ±4% if the true prevalence was between 5–10%. This sample size was also predicted to provide 90% power to detect an absolute difference of at least 15% in the prevalence of acute undernutrition between urban and rural areas if the prevalence in the urban area of Metro Manila was between 30% and 60%.

### Statistical methods

Data were analyzed using Stata, version 14.1 (College Station TX: StataCorp LP) and R^28^. Characteristics of those enrolled were summarized overall and by area (Metro Manila (urban) and Negros Occidental (rural)). Differences in characteristics between areas were tested with either a Fisher’s exact test, t-test or Wilcoxon rank sum test as appropriate for the data type. Prevalence of moderate or severe undernutrition, diabetes and moderate or severe anaemia are presented with corresponding 95% CIs overall and by area. Differences in prevalence of comorbidities by area were tested with Fisher’s exact tests. The distribution of HbA1c versus BMI by previous diagnosis status and by area are shown graphically, with associations between BMI and HbA1c tested using Spearman’s correlation coefficient. The extent of overlap between diabetes, moderate or severe undernutrition, moderate or severe anaemia and HIV are illustrated with Venn diagrams.

Associations with outcomes of undernutrition and diabetes were investigated using logistic regression. A multivariable model was built for each outcome using a forward stepwise approach, including factors associated with each outcome in univariable analyses based on a likelihood ratio test (LRT) p < 0.1, with retention and exclusion criteria of p < 0.1 from a likelihood ratio test comparing models at each model building step.

### Ethics

Ethical approval was obtained from the ethical review boards of The London School of Hygiene & Tropical Medicine (REF: 11995), The School of Tropical Medicine and Global Health, Nagasaki University (14^th^ October 2016) and the Philippines National Ethics Committee (REF: 2016–021-Cox-Malnutrition and Tuberculosis) and San Lazaro Hospital, Santa Cruz, Manila, the Philippines (23^rd^ February 2017). Written informed consent was obtained from all participants prior to study enrolment and additional written consent was obtained for optional HIV screening following Dept of Health procedures. All study procedures were conducted in accordance with UK, Japan and Filipino laws and regulations including the Philippines Data Privacy Act.

## Results

### Participants

A total of 637 persons were recruited into the study across the five sites; 338 (53.1%) from three urban sites (Metro Manila) and 299 (46.9%) from two rural sites (Table 1) during the period 8^th^ May to 18^th^ September 2017. This represented 63.4% of those identified from the registers in the rural sites and 73.2% in the urban sites. Of those enrolled, 37.9% (240/633) had a history of previous tuberculosis treatment, classified as either relapse, treatment after loss to follow-up, treatment failure or previous treatment outcome unknown. Thirty-five percent of participants had bacteriological confirmation (220/628) of tuberculosis disease. Compared to rural areas, participants in urban areas were notably younger (Fisher’s exact test test p < 0.001, mean age 41 years vs 50 years), more likely to be unmarried (Fisher’s exact test p < 0.001), more likely to have some level of education or training beyond primary education (Fisher’s exact test p < 0.001), not have health insurance (Fisher’s exact test p = 0.002), have a longer transportation time to the clinic (Fisher’s exact test p < 0.001), and less likely to be current smokers (Fisher’s exact test p < 0.001) or frequent drinkers (Fisher’s exact test p < 0.001). There was substantially more food insecurity reported in those enrolled at rural sites (Fisher’s exact test p < 0.001), compared to urban along with substantially more reported weight loss in the last 3–6 months (Fisher’s exact test p < 0.001) and severe decreases in food intake in the last month (Fisher’s exact test p < 0.001). In urban sites, slightly more participants were in the intensive rather than continuation phase of treatment (Fisher’s exact test p = 0.068). Only one study site (San Lazaro Hospital) in Metro Manila managed persons with MDR-TB and all those registered and alive were enrolled making up 10% of participants in the urban sites. There was no difference in the proportion of new cases between the urban and rural sites, but a higher rate of bacteriologically confirmed cases in the urban sites (Table 1).

**Table 1:** Number of patients per site and characteristics of enrolled TB DOTS patients.

		Metro Manila region (N = 338)	Negros Occidental region (N = 299)	Total (N = 637)	p-value^1^
Centre	San Nicolas	108			
	Pedro Gil	102			
	San Lazaro Hospital	128			
	Bago City		222		
	Valladolid		77		
Sex	Female	97 (28.7)	94 (31.4)	191 (30.0)	0.488
	Male	241 (71.3)	205 (68.6)	446 (70.0)	
Age (years)	18–40	183 (54.1)	89 (29.8)	272 (42.7)	<0.001
	41–65	127 (37.6)	154 (51.5)	281 (44.1)	
	>65	28 (8.3)	56 (18.7)	84 (13.2)	
Household size	mean (SD)	4.7 (3.1)	4.7 (2.2)	4.7 (2.7)	0.972
Marital status	Single	171 (50.6)	87 (29.1)	258 (40.5)	<0.001
	Married	132 (39.1)	165 (55.2)	297 (46.6)	
	Divorced/separated	8 (2.4)	6 (2.0)	14 (2.2)	
	Widowed	27 (8.0)	41 (13.7)	68 (10.7)	
Highest level of education obtained	Primary	63 (18.6)	155 (51.8)	218 (34.2)	<0.001
	Secondary	147 (43.5)	104 (34.8)	251 (39.4)	
	Tertiary	120 (35.5)	27 (9.0)	147 (23.1)	
	Vocational	8 (2.4)	13 (4.3)	21 (3.3)	
Health insurance^2^	No	145 (45.7)	98 (33.2)	243 (39.7)	0.002
	Yes	172 (54.3)	197 (66.8)	369 (60.3)	
Time to health clinic (minutes), median (range)		15 (0–300)	6 (1–120)	10 (0–300)	<0.001
Smoking status	No or Ex	304 (89.9)	209 (69.9)	513 (80.5)	<0.001
	Yes	34 (10.1)	90 (30.1)	124 (19.5)	
Alcohol consumption	Daily	25 (7.4)	92 (30.8)	117 (18.4)	<0.001
	Weekly or monthly	78 (23.1)	61 (20.4)	139 (21.8)	
	Rarely/Never	235 (69.5)	146 (48.8)	381 (59.8)	
Weight change last 3–6 months^3^	No change	70 (20.8)	32 (10.7)	102 (16.0)	<0.001
	Increase	73 (21.7)	18 (6.0)	91 (14.3)	
	Decrease	194 (57.6)	249 (83.3)	443 (69.7)	
Food intake last 1 month^3^	No change	196 (58.2)	43 (14.4)	239 (37.6)	<0.001
	Increase	66 (19.6)	79 (26.4)	145 (22.8)	
	Moderate decrease	65 (19.3)	98 (32.8)	163 (25.6)	
	Severe decrease	10 (3.0)	79 (26.4)	89 (14.0)	
Household Food security score	Secure	218 (64.5)	108 (36.1)	326 (51.2)	<0.001
	Moderate insecurity	69 (20.4)	98 (32.8)	167 (26.2)	
	Severe insecurity	51 (15.1)	93 (31.1)	144 (22.6)	
Multi-drug resistant TB	No	305 (90.2)	299 (100.0)	604 (94.8)	<0.001
	Yes	33 (9.8)	0 (0)	33 (5.2)	
Previous TB^4^	New case	206 (61.5)	187 (62.8)	393 (62.1)	0.806
	Relapse case	129 (38.5)	111 (37.3)	240 (37.9)	
Bacteriologically confirmed TB^5^	No	198 (60.2)	210 (70.2)	408 (65.0)	0.009
	Yes	131 (39.8)	89 (29.8)	220 (35.0)	
Treatment phase^6^	Intensive	199 (58.9)	133 (44.5)	332 (52.1)	<0.001
	Continuation	139 (41.1)	166 (55.5)	305 (47.9)	

Data are n (%) of non-missing values for each characteristic, unless otherwise indicated as mean (SD) or median (range).

^1^p-value from Fishers exact test for categorical variables, t-test to compare means or Wilcoxon rank sum test to compare medians.

^2^21 missing values in Metro Manila region and 4 missing values in Negros Occidental region.

^3^1 missing value in Metro Manila region.

^4^3 missing value in Metro Manila and 1 missing value in Negros Occidental region.

^5^9 missing values in Metro Manila region.

^6^Intensive treatment phase, unless extended, is the first 2 months of the 6 month standard TB-DOTS shorter regimen comprising isoniazid, rifampicin, pyrazinamide and ethambutol, followed by continuation phase comprising isoniazid and rifampicin only. The intensive phase of the 9/12 months WHO shorter regimen for multi-drug resistant TB is the first 4 months (unless extended) comprising kanamycin, moxifloxacin, prothionamide, clofazimine, pyrazinamide and high dose isoniazid and ethambutol followed by continuation phase comprising moxifloxacin, clofazimine, pyrazinamide and ethambutol only.

### Prevalence of co-morbidities

Amongst 634 individuals with BMI measurements, the prevalence of all undernutrition (BMI < 18.5 kg/m^2^) was 36.6% (95% CI: 32.8–40.5%), with 32.1% (95% CI: 27.2–37.4%) in the urban sites compared to 41.6% (95% CI: 36.0–47.4%) in the rural sites (Fisher’s exact test p = 0.017). The prevalence of moderate or severe undernutrition (BMI < 17 kg/m^2^) was 20.5% (95% CI: 17.4–23.9%) and more common in rural sites (Table 2, 25% vs 17%, Fisher’s exact test p = 0.014). The prevalence of moderate undernutrition (16 kg/m^2^ < BMI < 17 kg/m^2^) was 8.7% and the prevalence of severe undernutrition (BMI < 16 kg/m^2^) was 11.8%. (Table 2). Overall, 29 (4.6%) participants were overweight (BMI 25–30 kg/m^2^) and 6 (1.0%) were obese (BMI ≥ 30 kg/m^2^).

**Table 2:** Prevalence of co-morbidities amongst enrolled TB DOTS patients.

Characteristic		Metro Manila Region (N = 338)	Negros Occidental Region (N = 299)	Total	p-value^1^
Undernutrition^2^	None or mild	280/336, 83.3% (78.9–87.2%)	224/298, 75.2% (69.9–80.0%)	504/634, 79.5% (76.1–82.6%)	0.014
	Moderate or severe	56/336, 16.7% (12.8–21.1%)	74/298, 24.8% (20.0–30.1%)	130/634, 20.5% (17.4–23.9%)	
Undernutrition^2^	None	228/336, 67.9% (62.6–72.8%)	174/298, 58.4% (52.6–64.0%)	402/634, 63.4% (59.5–67.2%)	0.048
	Mild	52/336, 15.5% (11.8–19.8%)	50/298, 16.8% (12.7–21.5%)	102/634, 16.1% (13.3–19.2%)	
	Moderate	22/336, 6.5% (4.1–9.7%)	33/298, 11.1% (7.7–15.2%)	55/634, 8.7% (6.6–11.1%)	
	Severe	34/336, 10.1% (7.1–13.9%)	41/298, 13.8% (10.1–18.2%)	75/634, 11.8% (9.4–14.6%)	
Diabetes^3^	No	299/325, 92.0% (88.5–94.7%)	236/264, 89.4% (85.0–92.8%)	535/589, 90.8% (88.2–93.0%)	0.316
	Yes	26/325, 8.0% (5.3–11.5%)	28/264, 10.6% (7.2–15.0%)	54/589, 9.2% (7.0–11.8%)	
Anaemia^4^	None or mild	297/336, 88.4% (84.5–91.6%)	249/296, 84.1% (79.5–88.1%)	546/632, 86.4% (83.5–89.0%)	0.131
	Moderate or severe	39/336, 11.6% (8.4–15.5%)	47/296, 15.9% (11.9–20.5%)	86/632, 13.6% (11.0–16.5%)	
HIV^5^	No	229/303, 75.6% (70.3–80.3%)	39/41		–
	Yes	74/303, 24.4% (19.7–29.7%)	2/41		
Reported hypertension	No	312/338, 92.3% (88.9–94.9%)	270/299, 90.3% (86.4–93.4%)	582/637, 91.4% (88.9–93.4%)	0.398
	Yes	26/338, 7.7% (5.1–11.1%)	29/299, 9.7% (6.6–13.6%)	55/637, 8.6% (6.6–11.1%)	
Reported chronic obstructive pulmonary disease	No	338/338, 100.0% (98.9–100.0%)	296/299, 99.0% (97.1–99.8%)	634/637, 99.5% (98.6–99.9%)	0.103
	Yes	0/338, 0.0% (0.0–1.1%)	3/299, 1.0% (0.2–2.9%)	3/637, 0.5% (0.1–1.4%)	
Reported Chronic kidney disease	No	336/338, 99.4% (97.9–99.9%)	298/299, 99.7% (98.2–100.0%)	634/637, 99.5% (98.6–99.9%)	1.000
	Yes	2/338, 0.6% (0.1–2.1%)	1/299, 0.3% (0.0–1.8%)	3/637, 0.5% (0.1–1.4%)	
Inflammatory status (CRP)^6^	<5 mg/l	290/318, 91.2% (87.5–94.1%)	213/227, 93.8% (89.9–96.6%)	503/545, 92.3% (89.7–94.4%)	0.328
	≥5 mg/l	28/318, 8.8% (5.9–12.5%)	14/227, 6.2% (3.4–10.1%)	42/545, 7.7% (5.6–10.3%)	

^1^p-value from Fishers exact test for categorical variables.

^2^2 missing values in Metro Manila region and 1 missing value in Negros Occidental region. Undernutrition is defined using BMI kg/m^2^: mild: <18.5–17; moderate: <17–16; severe: <16; none: ≥18.5.

^3^13 missing values in Metro Manila region and 35 missing values in Negros Occidental region. Diabetes defined as HbA1c ≥ 6–5% or on current diabetes medication.

^4^2 missing values in Metro Manila region and 3 missing values in Negros Occidental region. Moderate or Severe anaemia defined as haemoglobin<10 g/dL.

^5^only 41/299 patients were tested for HIV in Negros Occidental region, 35 missing values in Metro Manila.

^6^C-REactive Protein; Ref. ^45^.

The prevalence of diabetes was 9.2% (95% CI: 7.0–11.8%) with 52% previously undiagnosed. The prevalence of severe or moderate anaemia was 13.6% (95% CI: 11.0–16.5%). The prevalence of self-reported hypertension was 8.6% (95% CI: 6.6–11.1). There was no evidence of a difference in prevalence between rural and urban areas for these conditions (Table 2).

In the urban sites (Metro Manila), HIV testing is a routine component of TB DOTS clinic enrolment. Of the 90% of urban participants whose HIV status was known, 74/303 (24%) were HIV positive. HIV status was unknown for 85% of participants in rural sites due to a low number providing consent for study-specific HIV testing (not a routine part of TB DOTS clinic enrolment), therefore subsequent analysis of data including HIV status is reported for urban areas only. Less than 1% of all enrolled participants reported previous doctor-diagnosed chronic obstructive lung disease or chronic kidney disease.

### Multimorbidity

**Figure 1:**
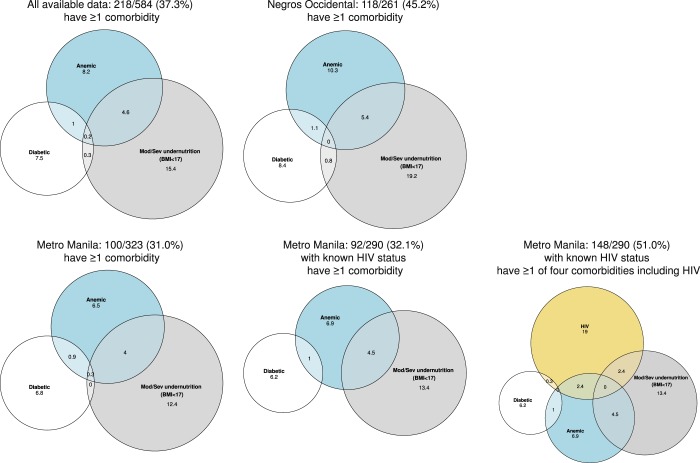
Percentage of persons with TB with co-morbidities^1^ overall and by area^1^ amongst participants with non-missing values for each comorbidity shown.

Approximately 40% of enrolled participants had at least one non-communicable clinical condition (moderate or severe undernutrition, diabetes and, or moderate or severe anaemia), as measured in 584 participants with non-missing data for each condition (Fig. 1, Supplementary Fig. 1). In urban sites 100/323 participants (31.0%) had at least one of these non-communicable conditions compared to 118/261 (45.2%) in rural sites (Fisher’s exact test p < 0.001). In urban sites, limited to those with known HIV status, the prevalence of at least one non-communicable condition was 32.1%. Fewer persons enrolled in urban sites were affected by one of the non-communicable conditions but 24.4% (74/303) of participants with known status had HIV-TB co-infection, only 8 of whom were newly diagnosed as the result of study screening. The most common combination of two conditions was undernutrition and anaemia in both the urban sites when limited to those with HIV data and in all participants in the rural sites (Supplementary Table 1).

### Diabetes in persons with TB disease

**Figure 2:**
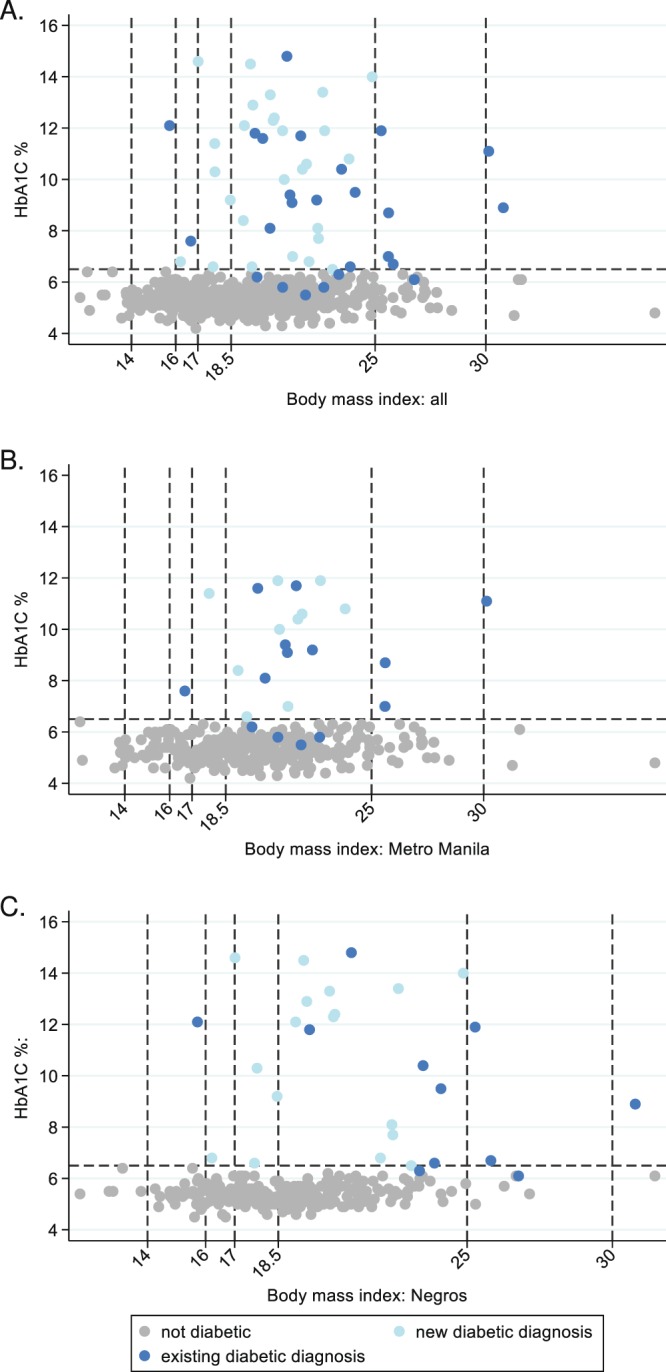
HbA1c by body mass index in new and previously diagnosed diabetes cases, overall and by urban/rural area.

Of 54 participants with diabetes, 26 (48%) reported a previous diagnosis of diabetes, 18 of whom (69%) reported regular diabetes follow-up visits at a health centre or with a doctor, whilst 24/26 (92%) reported currently taking diabetes medication (21 metformin or metformin + insulin). However, amongst those reporting diabetic medication, only 9 (38%) were controlled (HbA1c ≤7.0%) with HbA1c ranging to over 14% in those remaining (Fig. 2A). There was some evidence of higher HbA1c values in those with newly diagnosed versus previously diagnosed diabetes (median HbA1c in newly diagnosed = 10.5% and existing = 8.9%, Wilcoxon rank sum test p = 0.040, Fig. 2A). Although the majority of diabetes cases had normal BMI (38/54, 70.4%), or were overweight/obese (8/54, 14.8%), there were three diabetes cases in those with a BMI < 17 kg/m^2^, one of whom had severe undernutrition with a BMI < 16 kg/m^2^ (Fig. 2A). There was no apparent correlation between BMI and HbA1c levels (Fig. 2A–C) in those with previously diagnosed diabetes (Spearman p = 0.390), or newly diagnosed diabetes (Spearman p = 0.816). In participants with diabetes, hypertension was the most common second co-morbidity (11/54, 20.4%) with the majority (10/11) reporting current anti-hypertensive medication. This pattern of co-morbidity in diabetic participants was also observed when limited to those in the urban sites with complete data for all conditions including HIV (Supplementary Table 1).

### Factors associated with undernutrition

In univariable analyses (Table 3), participants were more likely to be undernourished if they were female (LRT p = 0.002), single or widowed compared to married (LRT p = 0.003), did not have health insurance (LRT p = 0.051), experienced weight loss in the previous 3–6 months (LRT p = 0.007), reported a moderate or severe reduction in food intake in the previous month (LRT p < 0.001), were classified as a relapse/treatment failure, loss to follow-up or previous treatment outcome unknown (LRT p = 0.004), were in the intensive versus continuation phase of TB treatment (LRT p = 0.037), had inflammation (CRP ≥ 5 mg/L; LRT p = 0.030), or were enrolled in the rural sites (LRT p = 0.011).

**Table 3:** Univariable and multivariable analysis of factors associated with undernutrition.

Characteristic		N	Undernourished, n (%)	Unadjusted OR (95% CI)	p-value^1^	Adjusted OR^2^ (95% CI)	p-value^1^
Sex	Female	190	54 (28.4)	1	0.002	1	0.001
	Male	444	76 (17.1)	0.52 (0.35df0.78)		0.44 (0.28–0.70)	
Age category (years)	18–40	271	47 (17.3)	1	0.216	—	
	41–65	279	65 (23.3)	1.45 (0.95–2.20)		—	
	>65	84	18 (21.4)	1.30 (0.71–2.39)		—	
Family size		634		0.96 (0.89–1.03)	0.282	—	
Marital status	Married	295	45 (15.3)	1	0.003	1	0.001
	Single	257	63 (24.5)	1.80 (1.18–2.76)		2.30 (1.44–3.67)	
	Divorced/separated	14	1 (7.1)	0.43 (0.05–3.35)		0.61 (0.07–4.95)	
	Widowed	68	21 (30.9)	2.48 (1.36–4.54)		2.29 (1.18–4.46)	
Highest level of education obtained	Primary	218	48 (22.0)	1	0.684	—	
	Secondary	249	53 (21.3)	0.96 (0.62–1.49)		—	
	Tertiary	146	25 (17.1)	0.73 (0.43–1.25)		—	
	Vocational	21	4 (19.0)	0.83 (0.27–2.59)		—	
Health insurance	No	242	60 (24.8)	1	0.051	—	
	Yes	368	67 (18.2)	0.68 (0.46–1.00)		—	
Time to health clinic (minutes)		634		1.00 (0.99–1.00)	0.281	—	
Smoking status	No or Ex	510	110 (21.6)	1	0.169	—	
	Yes	124	20 (16.1)	0.70 (0.41–1.18)		—	
Alcohol consumption	Daily	117	28 (23.9)	1	0.503	—	
	Weekly or monthly	139	25 (18.0)	0.70 (0.38–1.28)		—	
	Rarely/Never	378	77 (20.4)	0.81 (0.50–1.33)		—	
Weight change last 3–6 months	No change	102	16 (15.7)	1	0.007	—	
	Increase	91	10 (11.0)	0.66 (0.28–1.55)		—	
	Decrease	441	104 (23.6)	1.66 (0.93–2.95)		—	
Food intake last 1 month	No change	238	33 (13.9)	1	<0.001	1	<0.001
	Increase	145	24 (16.6)	1.23 (0.70–2.18)		1.47 (0.79–2.72)	
	Moderate decrease	163	42 (25.8)	2.16 (1.30–3.58)		2.41 (1.39–4.17)	
	Severe decrease	88	31 (35.2)	3.38 (1.91–5.98)		4.48 (2.40–8.37)	
Household Food security	Secure	324	57 (17.6)	1	0.147	—	
	Moderate insecurity	167	37 (22.2)	1.33 (0.84–2.12)		—	
	Severe insecurity	143	36 (25.2)	1.58 (0.98–2.53)		—	
TB treatment phase1	Intensive	329	78 (23.7)	1	0.037	—	
	Continuation	305	52 (17.0)	0.66 (0.45–0.98)		—	
Previous TB	New case	393	67 (17.1)	1	0.005	1	0.002
	Relapse case	237	63 (26.6)	1.76 (1.19–2.60)		1.97 (1.28–3.04)	
Bacteriologically confirmed TB	No	407	74 (18.2)	1	0.072	1	0.050
	Yes	218	53 (24.3)	1.45 (0.97–2.15)		1.56 (1.00–2.42)	
Inflammatory status (CRP)^3^	<5 mg/l	502	96 (19.1)	1	0.030	—	
	≥5 mg/l	41	14 (34.2)	2.19 (1.11–4.34)		—	
Area	Urban	336	56 (16.7)	1	0.011	—	
	Rural	298	74 (24.8)	1.65 (1.12–2.44)		—	

OR = odds ratio, CI = confidence interval. ^1^p-value from likelihood ratio test.

^2^Adjusted ORs shown for covariates retained in the final multivariable model (if effects are not reported, covariate was not included).

^3^C-reactive protein Ref. ^45^.

In a final multivariable model (Table 3), reduced food intake reported in the previous month, phase of TB treatment, marital status and sex, remained associated with undernutrition after adjustment. After adjustment for these four variables, none of; weight change in the previous 3–6 months (LRT p = 0.190), health insurance (LRT p = 0.165) or area (LRT p = 0.241) were associated with undernutrition and so were not retained in the final model. Differences by area observed in Table 2 were mainly explained by reduced food intake in the previous month being more common in the rural sites. In a reduced dataset due to missing data, inflammation was not associated (LRT p = 0.129) with undernutrition after adjustment for food intake in the previous month, phase of TB treatment, marital status and sex. There was no evidence of interaction between sex and any of the other covariates in the final model (LRT p > 0.1). Fitting the final model in just the urban sites where HIV was tested and those with MDR-TB were enrolled, there was no evidence of increased odds of undernutrition in HIV positive versus negative participants (adjusted odds ratio, AOR = 0.54; 95% CI: 0.22–1.34, LRT p = 0.177), or in those with MDR-TB versus drug sensitive TB (AOR = 1.66, 95% CI: 0.69–4.01, LRT p = 0.274).

### Undernutrition and anaemia in persons with drug sensitive TB

Although the prevalence of moderate/severe undernutrition was lower in persons in the continuation phase compared to intensive phase of TB treatment (Table 3), a relatively high prevalence was still observed in persons nearing completion of their DS-TB treatment in the 5^th^ or 6th month at 13.9% (26/187). Similarly, the prevalence of moderate and severe anaemia was also lower in persons in continuation compared to intensive phase of treatment (9.6% *vs*. 17.3% Fisher’s exact test p = 0.005). However, in months 5 or 6 of drug sensitive treatment when TB/inflammatory associated anaemia is expected to have resolved it was observed that of 186 participants: 29 (15.6%) had mild, 14 (7.5%) moderate and 2 (1.1%) had severe anaemia. Of 15 cases of moderate/severe anaemia in month 5 or more of treatment and with CRP measurements, only 3 (20%) had inflammation (CRP > 5 mg/L). Thus, anaemia in the final months of treatment did not appear to be fully explained by remaining inflammation, suggesting a role for iron deficiency in these cases of anaemia.

### Factors associated with diabetes

In univariable analyses, increasing age (LRT p < 0.001), having health insurance (LRT p = 0.051), being married or widowed rather than being single (LRT p = 0.003), and central obesity (waist-to-hip ratio >0.9 for men and >0.85 for women^26^) (LRT p < 0.001) were associated with having existing or newly diagnosed diabetes (Table 4). BMI categorization into normal, underweight, overweight or obese was associated with odds of diabetes (LRT p < 0.001), mostly due to the odds of diabetes being much lower in those who were undernourished compared to those with normal weight. Few participants were overweight or obese (n = 34) and whilst odds of diabetes were higher than for those with normal weight, these did not reach statistical significance. Marital status was no longer associated with diabetes after adjustment for age (LRT p = 0.680) and the final multivariable model included age group, health insurance and central obesity based on waist-to-hip ratio (Table 4). Fitting the final model in just the urban sites including HIV status and participants with MDR-TB were enrolled, there was no evidence of increased odds of diabetes in HIV positive versus negative persons (AOR = 0.21, 95% CI: 0.02–1.98, LRT p = 0.126), or in those with MDR-TB versus drug sensitive TB (AOR = 2.91, 95% CI: 0.85–10.0, LRT p = 0.103).

**Table 4:** Univariable and multivariable analysis of factors associated with Type 2 diabetes.

Characteristic		N	Type 2 diabetes, n (%)	Unadjusted OR (95% CI)	p-value^1^	Adjusted OR^2^ (95% CI)	p-value^1^
Sex	Female	173	17 (9.8)	1	0.723	—	
	Male	416	37 (8.9)	0.90 (0.49–1.64)		—	
Age category (years)	18–40	256	6 (2.3)	1	<0.001	1	<0.001
	41–65	258	41 (15.9)	7.87 (3.28–18.9)		5.93 (2.41–14.6)	
	>65	75	7 (9.3)	4.29 (1.40–13.2)		3.07 (0.97–9.70)	
Family size		589		0.93 (0.83–1.05)	0.219	—	
Marital status	Single	243	10 (4.1)	1	0.003	—	
	Married	273	36 (13.2)	3.54 (1.72–7.30)		—	
	Divorced/separated	11	1 (9.1)	2.33 (0.27–20.0)		—	
	Widowed	62	7 (11.3)	2.97 (1.08–8.14)		—	
Highest level of education obtained	Primary	197	16 (8.1)	1	0.221	—	
	Secondary	231	28 (12.1)	1.56 (0.82–2.98)		—	
	Tertiary	142	9 (6.3)	0.77 (0.33–1.79)		—	
	Vocational	19	1 (5.3)	0.63 (0.08–5.02)		—	
Health insurance	No	226	14 (6.2)	1	0.051	1	0.032
	Yes	350	38 (10.9)	1.84 (0.98–3.49)		2.05 (1.05–4.05)	
Time to health clinic (minutes)		589		0.99 (0.98–1.00)	0.128	—	
Smoking status	No or Ex	476	44 (9.2)	1	0.896	—	
	Yes	113	10 (8.8)	0.95 (0.46–1.96)		—	
Alcohol consumption	Daily	111	12 (10.8)	1	0.783	—	
	Weekly or monthly	133	11 (8.3)	0.74 (0.31–1.76)		—	
	Rarely/Never	345	31 (9.0)	0.81 (0.40–1.65)		—	
Weight change last 3–6 months	No change	97	8 (8.2)	1	0.730	—	
	Increase	82	6 (7.3)	0.88 (0.29–2.64)		—	
	Decrease	410	40 (9.8)	1.20 (0.54–2.66)		—	
Food intake last 1 month	No change	223	18 (8.1)	1	0.553	—	
	Increase	136	10 (7.4)	0.90 (0.40–2.02)		—	
	Moderate decrease	152	17 (11.2)	1.43 (0.71–2.88)		—	
	Severe decrease	78	9 (11.5)	1.49 (0.64–3.46)		—	
Food security	Secure	308	30 (9.7)	1	0.689	—	
	Moderate insecurity	148	11 (7.4)	0.74 (0.36–1.53)		—	
	Severe insecurity	133	13 (9.8)	1.00 (0.51–1.99)		—	
TB treatment phase	Intensive	307	32 (10.4)	1	0.269	—	
	Continuation	282	22 (7.8)	0.73 (0.41–1.28)		—	
Previous TB	New case	366	31 (8.5)	1	0.523	—	
	Relapse case	219	22 (10.1)	1.21 (0.68–2.14)		—	
Bacteriologically confirmed TB	No	373	29 (7.8)	1	0.136	—	
	Yes	208	24 (11.5)	1.55 (0.88–2.74)		—	
Inflammatory status (CRP)	<5 g/l	502	44 (8.8)	1	0.121	—	
	≥5 g/l	42	7 (16.7)	2.08 (0.87–4.96)		—	
BMI group^3^	Normal	216	8 (3.7)	1	<0.001	—	
	Underweight	337	38 (11.3)	0.30 (0.14–0.66)		—	
	Overweight	28	6 (21.4)	2.15 (0.82–5.63)		—	
	Obese	6	2 (33.3)	3.93 (0.70–22.2)		—	
Central obesity: waist-to-hip ratio^4^	No	393	13 (3.3)	1	<0.001	1	<0.001
	Yes	195	41(21.0)	7.78 (4.06–14.9)		6.16 (3.15–12.0)	
Area	Urban	325	26 (8.0)	1	0.277	—	
	Rural	264	28 (10.6)	1.36 (0.78–2.39)		—	

OR = odds ratio, CI = confidence interval. ^1^p-value from likelihood ratio test.

^2^Adjusted ORs shown for covariates retained in the final multivariable model (if effects are not reported, covariate was not included).

^3^underweight: Body Mass Index underweight: <18.5, normal: 18.5–24.9, overweight: 25.0–29.9, obese ≥30 kg/m^2^.

^4^based on waist-to-hip ratio >0.85 for women and >0.9 for men.

Given the strong association between central obesity based on waist-to-hip ratio cut-off value of >0.85 for women and >0.9 for men, an analysis was conducted to assess the sensitivity and specificity of different waist-to-hip ratio thresholds to correspond to diabetes. Thresholds were considered within a range of 0.7–1.1 in increments of 0.05. Results of these simple, unadjusted analyses suggest a threshold of >0.85 for persons of both sexes in this study would have sensitivity of 87% (95% CI: 0.75–95%) and specificity of 54% (95% CI: 50–58%, Supplementary Table 2).

## Discussion

The aim of this cross-sectional study was to evaluate the burden and predictors of diabetes, undernutrition and anaemia co-morbidities in TB DOTS outpatients in a rural and an urban setting in the Philippines and to describe co-morbidity inter-relationships including with HIV-infection. To our knowledge, this study provides the first published estimates of the prevalence of malnutrition and diabetes among persons receiving anti-TB treatment (ATT) in routine outpatient TB-DOTs clinics in the Philippines. The prevalence of diabetes, at 9.1%, with more than 50% of diabetes cases newly diagnosed by the study screening was higher than current national estimates for the Philippines general population (6.1%,WHO 2016)^29^. but lower than the 14.1% reported in 634 urban HIV-negative Indonesians with newly diagnosed pulmonary TB, systematically assessed for diabetes by fasting blood glucose^30^ and more recently of 19.7% in the Indonesian participants of the multi-country “TANDEM study”^31^. Previous limited reports in the Philippines include a diabetes prevalence of 18% in pulmonary TB patients in Quezon City (N = 38)^32^, and 17.7% in a cohort of acutely unwell admissions to a TB ward in Manila (N = 348)^33^.

Undernutrition (BMI < 18.5 kg/m^2^) was the most common comorbidity affecting just over a third of all participants and was more common in the rural sites where greater household food insecurity, decreased food intake and recent weight loss were also reported. There was relatively little clustering of multimorbidity within individuals, with surprisingly little overlap between moderate/severe undernutrition and moderate/severe anaemia or between HIV and non-communicable conditions, for which HIV is a known risk factor. On further exploration, the participants in urban areas living with HIV were younger than those without HIV (31.7 vs 44.4 years) and only 2 were new diagnoses not already enrolled in HIV care programmes, which may explain these observations.

The high prevalence of moderate and severe undernutrition, including in the final months of treatment, when possible TB-mediated catabolic effects should have resolved, combined with frequent household food insecurity, indicate an important unmet need for nutritional support in persons with TB. Similar findings have been reported in a rural TB cohort in India, but at a higher prevalence and degree of severity, with 47% of males and 62% of females being severely undernourished (BMI < 16 kg/m^2^) at the start of treatment and 17% of males and 35% of females at treatment completion. The prevalence of severe anaemia (haemoglobin <8 g/dL) at start of treatment was 20%, whilst diabetes was reported in 11%. HIV prevalence was 4.4%. Unfortunately, this study did not report the proportions of participants with multiple co-morbidities^34^. The WHO guideline on Nutritional care and support for patients with tuberculosis states that “because of the clear bidirectional causal link between undernutrition and active TB, nutrition screening, assessment and management are integral components of TB treatment and care”^35^. These study results can be utilized in the Philippines to inform planning of TB treatment and care related to undernutrition, in which currently height is not assessed to allow calculation of BMI and no anaemia screening occurs. Many countries offer cash transfers or some kind of nutritional support to those undergoing treatment for MDR-TB, but do not extend this to drug sensitive TB (including the Philippines). Participants with a previous history of TB were almost twice as likely to be undernourished, indicating a probable downward spiral effect of undernutrition and TB. Thus, not addressing undernutrition during TB treatment may be contributing to the high rates of TB recurrence (38% of participants reported previous TB disease and were classified as relapse, treatment failure, loss to follow-up or previous treatment unknown). It remains to be determined if effectively treating undernutrition during an initial TB treatment can reduce risk of TB recurrence and thus be a cost-effective intervention. In this outpatient population, men had a lower risk of undernutrition than women. This is in contrast to our observation in a Filipino inpatient cohort (also 70% male) in whom there was no difference in the prevalence of moderate/severe undernutrition by sex, but in whom there was a statistically significant difference in the risk of inpatient death between male and female undernourished patients, with a greater risk in men^33^.

Poor glucose control is associated with poorer treatment outcomes^36–38^ (reviewed in^39^), and increased risk of developing diabetes associated complications and recurrent TB^40^. Although there is currently limited direct evidence that improved glucose control improves TB treatment outcomes, the high prevalence of poorly controlled blood glucose; even when using the more realistic target of HbA1c% <8%^40^, and including in those reporting to be on regular diabetes medication is worrying. The data suggests that both tuberculosis and non-communicable disease programmes have a large task of ensuring glycemic control among their program clients. The risk of diabetes was the greatest in the middle-aged group (40–65 years). However, unlike recent data from a multi-country study investigating diabetes in TB (the TANDEM study), we did not observe an association between diabetes and previous TB or with smear positive cases^31^. The distribution of HbA1c values for our diabetic population (new and previously diagnosed cases) was also similar to that from the Indonesian TANDEM study population, in whom a single point of care (POC) HbA1c test was shown to have high sensitivity and specificity to detect new diabetes cases, adding confidence to our diagnoses of diabetes using this single POC measurement^41^.

Our data demonstrate that diabetes whether previously or newly diagnosed was not limited to those with higher BMI, and could also occur in those with severe or moderate undernutrition. In multivariable analysis, waist to hip ratio was strongly associated with diabetes, whilst BMI was not significant. We thus investigated how, in resource-limited settings, waist-to-hip ratio might be useful as a screening tool for prioritising individuals for diabetes testing and observed a high sensitivity of 87% for a single cut-off for both sexes, supporting its potential use, at least in the Filipino context. Mean waist to hip ratio was also significantly increased in the TANDEM study, and most significantly in Indonesia^31^, although neither waist to hip ratio or waist circumference were included in the final multicountry predictive risk score for diabetes in persons with TB^41^.

TB programmes, in which individuals are in regular contact with local health services, provide an opportunity to increase rates of diabetes diagnosis (and other important NCD conditions like hypertension), and to re-engage with persons with diabetes who may have dropped out of diabetes care. Although there is still limited evidence to support how persons with TB-diabetes should be managed and how integration of services can be achieved, specific guidelines on management have recently been released based on best available evidence and expert opinion^42^. Diabetes management should include drug management, nutritional status assessment, dietary and lifestyle counselling (healthy diet, weight management, physical activity, smoking cessation and avoiding excess alcohol), which are also recommended as part of routine TB care. Training of TB health workers in diagnosing and managing diabetes, undernutrition and anaemia would provide a more holistic and patient-centered approach to TB management and hopefully avoid conflicting advice being given. Integration of services, which are currently split between infectious and non-infectious, will present significant challenges but also opportunities for leveraging of resources and investments between domestic and international funding sources.

This study did not rely on routine testing or self-reporting of diabetes or undernutrition and used trained research nurses to conduct all assessments and anthropometry. Although the use of a single POC test for HbA1c is not currently recommended for diabetes diagnosis by regulatory bodies, its agreement with accredited laboratory measurement using high performance liquid chromatography in persons with TB has recently been shown to be high, with minimal effects on clinical misclassification^43^. However, the higher proportion of participants with severe anaemia in our setting may have resulted in some misclassification in this group. It has been reported that severe anaemia can affect HbA1c, with possible over-estimation in iron deficiency anaemia and under-estimation with haemolytic anaemia^44^. In the small number of those with severe anaemia in the study comparing POC HbA1c with laboratory HbA1c, the POC test showed a mean difference of +1.1%^43^. The amount of missing information for HIV status is a limitation, especially for the rural sites. HIV in TB in the Philippines is still low at 2%^1^. The prevalence reported in the urban sites of this study is not representative of the majority of Filipino persons with TB due to the inclusion of a hospital site which is one of the largest HIV referral sites in the city. This can be seen as another limitation, but also allowed us, with a reasonable study sample size, to explore the effect of HIV on the frequency and overlap of our other clinical conditions of interest. Finally, hypertension was not directly assessed but was based on patient report. Our diabetes prevalence estimate could be overestimated due to TB induced transient hyperglycemia, greater in the early phase of treatment; or underestimated if those with diabetes were less likely to be included due to more frequent poor TB treatment outcomes, or were of higher social status and less likely to be treated for TB in public facilities. The cross-sectional study design thus limits the conclusions that can be drawn regarding possible causality between changes in the prevalence of co-morbid conditions over TB treatment duration.

Future studies should directly assess hypertension, other respiratory conditions and diabetes associated co-morbidities such as peripheral neuropathy, retinopathy, kidney function and blood lipids to further inform specific management strategies and resources for diabetes in management in TB programmes. The evidence base also requires further strengthening, in relation to context-specific dietary management strategies, especially of diabetes in those who are also undernourished, and of the effects of nutritional supplementation on TB, multimorbidity and long-term health sequalae of TB.

## Conclusions

This study provides evidence of a high burden of co-morbidities in Filipino persons with drug-sensitive TB, including in rural areas. In this study, multimorbidity was not a problem limited to those with HIV co-infection. Moderate and severe undernutrition and anaemia were also observed in the final months of TB treatment indicating the need for additional interventions to detect and treat these conditions. Our findings are likely applicable to other country settings with a double burden of under-and overnutrition and low HIV. For effective management of multimorbidity, health care programmes need to move towards integration of services and include support for nutrition and lifestyle management.

## Supplementary information

Supplementary Information.

## Data Availability

The complete dataset and associated meta-data and data dictionary representing the data generated and analysed during the current study are available in the FigShare repository: 10.6084/m9.figshare.11695467. The study and protocol are registered at **ISRCTN12506117**.
