# Supplementary material for: Patterns and predictors of co-morbidities in Tuberculosis: A cross-sectional study in the Philippines
Source: Sci Rep. 2020 Mar 5;10:4100. doi: 10.1038/s41598-020-60942-2 (PMC7058028; doi:10.1038/s41598-020-60942-2)
Supplement: Supplementary file 1 — Supplementary Information. [file 41598_2020_60942_MOESM1_ESM.pdf]

# Supplementary Information:

# Patterns and predictors of co-morbidities in Tuberculosis: A cross-sectional study in the Philippines

Laura V White, Tansy Edwards, Nathaniel Lee, Mary C Castro, Naomi R Saludar, Rugaiya W Calapis, Benjamin N Faguer, Nelson Dela Fuente, Ferdinand Mayoga, Nobuo Saito, Koya Ariyoshi, Anna Marie Celina G Garfin, Juan A Solon & Sharon E Cox.

**Supplementary Table 1. Prevalence and pattern of co-morbidities by area in complete dataset including HIV and reported hypertension.**

| Number of comorbidities | Undernutrition | Diabetes | Moderate/severe anaemia | Reported hypertension | HIV | n (%) |
|---|---|---|---|---|---|---|
| Urban/Metro Manila[1]: N=290 | | | | | | |
| 0 | No | No | No | No | No | 128 (44.1) |
| 1 | No | No | No | No | Yes | 54 (18.6) |
| 1 | Yes | No | No | No | No | 32 (11.0) |
| 1 | No | No | No | Yes | No | 14 (4.8) |
| 1 | No | Yes | No | No | No | 13 (4.5) |
| 1 | No | No | Yes | No | No | 11 (3.8) |
| 2 | Yes | No | Yes | No | No | 8 (2.8) |
| 2 | Yes | No | No | No | Yes | 7 (2.4) |
| 2 | No | No | Yes | No | Yes | 7 (2.4) |
| 2 | No | Yes | No | Yes | No | 4 (1.4) |
| 2 | No | Yes | Yes | No | No | 3 (1.0) |
| 2 | No | No | Yes | Yes | No | 2 (0.7) |
| 2 | No | Yes | No | No | Yes | 1 (0.3) |
| 2 | No | No | No | Yes | Yes | 1 (0.3) |
| 3 | Yes | No | Yes | No | Yes | 4 (1.4) |
| 3 | Yes | No | Yes | Yes | No | 1 (0.3) |
| Rural/Negros Occidental[1]: N=261 | | | | | | |
| 0 | No | No | No | No | - | 130 (44.8) |
| 1 | Yes | No | No | No | - | 47 (16.2) |
| 1 | No | No | Yes | No | - | 23 (7.9) |
| 1 | No | Yes | No | No | - | 16 (5.5) |
| 1 | No | No | No | Yes | - | 13 (4.5) |
| 2 | Yes | No | Yes | No | - | 13 (4.5) |
| 2 | No | Yes | No | Yes | - | 6 (2.1) |
| 2 | No | No | Yes | Yes | - | 4 (1.4) |
| 2 | Yes | No | No | Yes | - | 3 (1.0) |
| 2 | Yes | Yes | No | No | - | 2 (0.7) |
| 2 | No | Yes | Yes | No | - | 2 (0.7) |
| 3 | No | Yes | Yes | Yes | - | 1 (0.3) |
| 3 | Yes | No | Yes | Yes | - | 1 (0.3) |

[1] number of participants with non-missing values for each co-morbidity shown

**Supplementary Table 2. Waist-to-hip ratio as a predictor of diabetes**

| Waist-to ratio threshold | Sensitivity | Specificity | Positive predictive value | Negative predictive value | False negative rate |
|---|---|---|---|---|---|
| All: | | | | | |
| 0.70 | 1.00 (0.93 - 1.00) | 0.01 (0.00 - 0.02) | 0.09 (0.07 - 0.12) | 1.00 (0.48 - 1.00) | 0.00 (0.00 - 0.07) |
| 0.75 | 1.00 (0.93 - 1.00) | 0.06 (0.04 - 0.09) | 0.10 (0.07 - 0.13) | 1.00 (0.90 - 1.00) | 0.00 (0.00 - 0.07) |
| 0.80 | 0.96 (0.87 - 1.00) | 0.25 (0.22 - 0.29) | 0.12 (0.09 - 0.15) | 0.99 (0.95 - 1.00) | 0.04 (0.00 - 0.13) |
| **0.85** | **0.87 (0.75 - 0.95)** | **0.54 (0.50 - 0.58)** | **0.16 (0.12 - 0.21)** | **0.98 (0.95 - 0.99)** | **0.13 (0.05 - 0.25)** |
| 0.90 | 0.59 (0.45 - 0.72) | 0.78 (0.74 - 0.82) | 0.21 (0.15 - 0.29) | 0.95 (0.93 - 0.97) | 0.41 (0.28 - 0.55) |
| 0.95 | 0.28 (0.16 - 0.42) | 0.93 (0.90 - 0.95) | 0.28 (0.17 - 0.42) | 0.93 (0.90 - 0.95) | 0.72 (0.58 - 0.84) |
| 1.00 | 0.07 (0.02 - 0.18) | 0.99 (0.97 - 0.99) | 0.33 (0.10 - 0.65) | 0.91 (0.89 - 0.93) | 0.93 (0.82 - 0.98) |
| 1.05 | 0.00 (0.00 - 0.07) | 0.99 (0.98 - 1.00) | 0.00 (0.00 - 0.71) | 0.91 (0.88 - 0.93) | 1.00 (0.93 - 1.00) |
| 1.10 | 0.00 (0.00 - 0.07) | 0.99 (0.98 - 1.00) | 0.00 (0.00 - 0.71) | 0.91 (0.88 - 0.93) | 1.00 (0.93 - 1.00) |
| Female: | | | | | |
| 0.70 | 1.00 (0.80 - 1.00) | 0.03 (0.01 - 0.06) | 0.10 (0.06 - 0.16) | 1.00 (0.40 - 1.00) | 0.00 (0.00 - 0.20) |
| 0.75 | 1.00 (0.80 - 1.00) | 0.17 (0.11 - 0.23) | 0.12 (0.07 - 0.18) | 1.00 (0.87 - 1.00) | 0.00 (0.00 - 0.20) |
| 0.80 | 1.00 (0.80 - 1.00) | 0.40 (0.32 - 0.48) | 0.15 (0.09 - 0.23) | 1.00 (0.94 - 1.00) | 0.00 (0.00 - 0.20) |
| **0.85** | **0.94 (0.71 - 1.00)** | **0.68 (0.60 - 0.75)** | **0.24 (0.15 - 0.36)** | **0.99 (0.95 - 1.00)** | **0.06 (0.00 - 0.29)** |
| 0.90 | 0.59 (0.33 - 0.82) | 0.84 (0.77 - 0.89) | 0.29 (0.15 - 0.46) | 0.95 (0.90 - 0.98) | 0.41 (0.18 - 0.67) |
| 0.95 | 0.29 (0.10 - 0.56) | 0.96 (0.92 - 0.99) | 0.45 (0.17 - 0.77) | 0.93 (0.87 - 0.96) | 0.71 (0.44 - 0.90) |
| 1.00 | 0.06 (0.00 - 0.29) | 0.97 (0.94 - 0.99) | 0.20 (0.01 - 0.72) | 0.90 (0.85 - 0.94) | 0.94 (0.71 - 1.00) |
| 1.05 | 0.00 (0.00 - 0.20) | 0.99 (0.96 - 1.00) | 0.00 (0.00 - 0.98) | 0.90 (0.85 - 0.94) | 1.00 (0.80 - 1.00) |
| 1.10 | 0.00 (0.00 - 0.20) | 0.99 (0.96 - 1.00) | 0.00 (0.00 - 0.98) | 0.90 (0.85 - 0.94) | 1.00 (0.80 - 1.00) |
| Male: | | | | | |
| 0.70 | 1.00 (0.91 - 1.00) | 0.00 (0.00 - 0.01) | 0.09 (0.06 - 0.12) | 1.00 (0.03 - 1.00) | 0.00 (0.00 - 0.09) |
| 0.75 | 1.00 (0.91 - 1.00) | 0.02 (0.01 - 0.04) | 0.09 (0.06 - 0.12) | 1.00 (0.63 - 1.00) | 0.00 (0.00 - 0.09) |
| 0.80 | 0.95 (0.82 - 0.99) | 0.19 (0.15 - 0.24) | 0.10 (0.07 - 0.14) | 0.97 (0.91 - 1.00) | 0.05 (0.01 - 0.18) |
| **0.85** | **0.84 (0.68 - 0.94)** | **0.48 (0.43 - 0.53)** | **0.14 (0.09 - 0.19)** | **0.97 (0.93 - 0.99)** | **0.16 (0.06 - 0.32)** |
| 0.90 | 0.59 (0.42 - 0.75) | 0.76 (0.71 - 0.80) | 0.19 (0.13 - 0.28) | 0.95 (0.92 - 0.97) | 0.41 (0.25 - 0.58) |
| 0.95 | 0.27 (0.14 - 0.44) | 0.92 (0.88 - 0.94) | 0.24 (0.12 - 0.39) | 0.93 (0.90 - 0.95) | 0.73 (0.56 - 0.86) |
| 1.00 | 0.08 (0.02 - 0.22) | 0.99 (0.97 - 1.00) | 0.43 (0.10 - 0.82) | 0.92 (0.89 - 0.94) | 0.92 (0.78 - 0.98) |
| 1.05 | 0.00 (0.00 - 0.09) | 0.99 (0.98 - 1.00) | 0.00 (0.00 - 0.84) | 0.91 (0.88 - 0.94) | 1.00 (0.91 - 1.00) |
| 1.10 | 0.00 (0.00 - 0.09) | 0.99 (0.98 - 1.00) | 0.00 (0.00 - 0.84) | 0.91 (0.88 - 0.94) | 1.00 (0.91 - 1.00) |

**Supplementary Figure 1. Number of persons with TB with co-morbidities*, overall and by area**

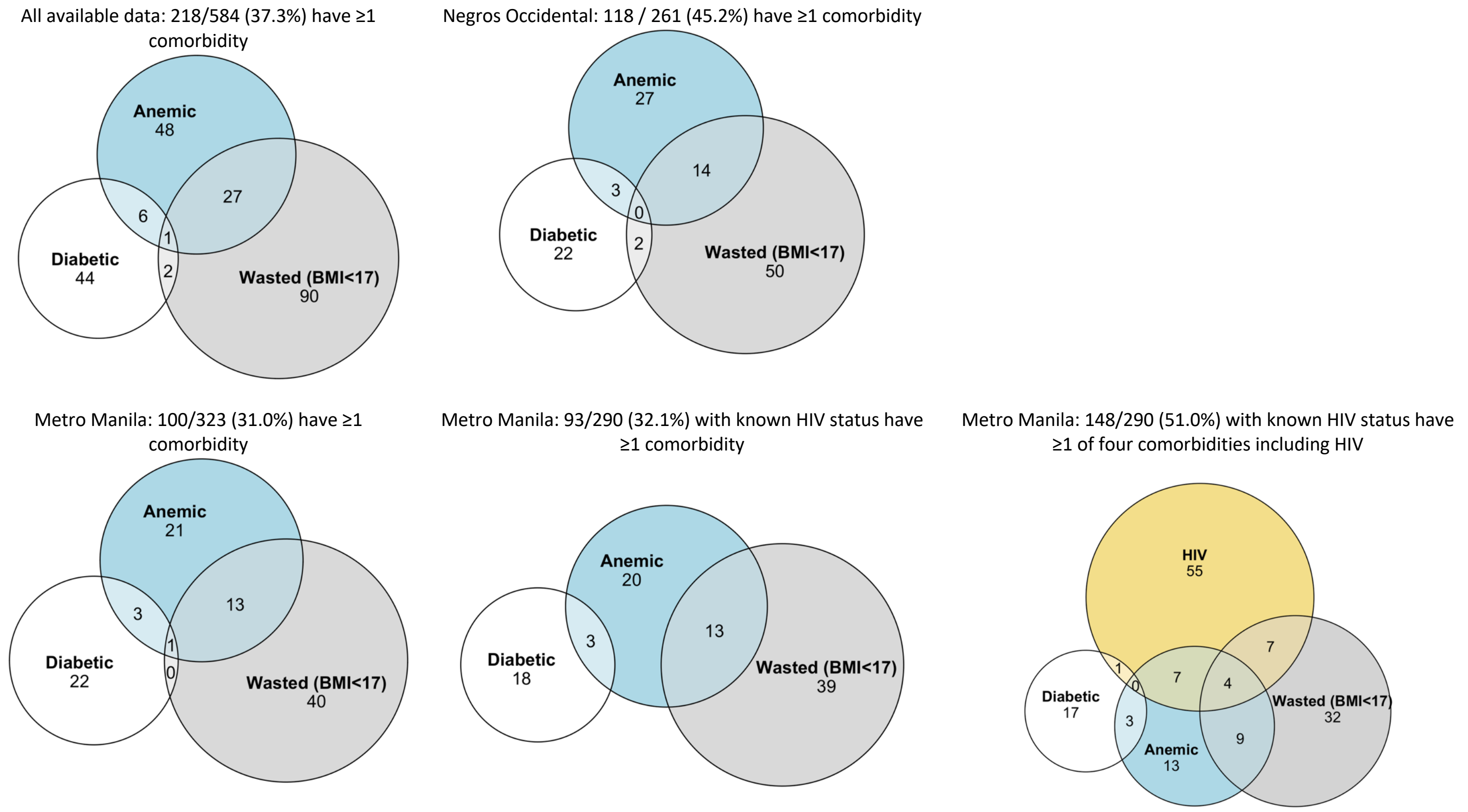


* amongst participants with non-missing values for each comorbidity shown
